# A Rare Case of Pyometra in a 65-Year-Old Post-menopausal Woman

**DOI:** 10.7759/cureus.64973

**Published:** 2024-07-20

**Authors:** Rishabh Dhabalia, Pratapsingh Parihar, Shivali V Kashikar, Suhit Naseri, Shakti Sagar

**Affiliations:** 1 Radiodiagnosis, Jawaharlal Nehru Medical College, Datta Meghe Institute of Medical Sciences (DMIMS), Wardha, IND; 2 Pathology, Datta Meghe Institute of Medical Sciences (DMIMS) School of Epidemiology and Public Health, Wardha, IND; 3 Pathology, Datta Meghe Institute of Higher Education and Research, Wardha, IND

**Keywords:** intrauterine infection, mri, endometrial collection, postmenopausal, pyometra

## Abstract

Pyometra is a gynecological condition characterized by pus accumulation in the endometrial cavity. It is a rare condition, and it should be included in the differential diagnosis of abdominal pain in postmenopausal women. We present a case of a 65-year-old postmenopausal woman with complaints of foul-smelling white discharge, itching in the perineal region, lower abdominal pain, and postmenopausal bleeding for two to three months. USG of the pelvis was done outside, which revealed heterogeneous ill-defined cervical growth with endometrial fluid collection and multiple uterine fibroids. CT and MRI of the pelvis were done in our hospital, which revealed an ill-defined heterogeneously enhancing growth in the cervix with multiple uterine fibroids and heterogeneous endometrial collection showing restricted diffusion in MRI suggestive of pyometra. Cervical biopsy revealed features suggestive of moderately differentiated squamous cell carcinoma.

## Introduction

Pyometra is a rare gynecological condition defined as an accumulation of purulent fluid within the endometrial cavity [1,2]. Pyometra develops as a result of increased production of progesterone. Increased progesterone production leads to cervical canal enlargement, allowing local microorganisms to migrate retrogradely through the cervix from the vagina into the uterine cavity [3]. The most common risk factors include post-surgical, post-intrauterine device (IUD) placement or removal, post-partum, and post-menopausal women [4]. The causes of pyometra include cervical or uterine malignancy, endometrial polyps, endometritis/pelvic inflammatory disease, cervical stenosis (most commonly post-operative), and structural anomalies (imperforate hymen) [1,5]. Patients usually present with post-menopausal bleeding, white discharge, fever, and abdominal pain. Some patients might even be asymptomatic [6]. The overall incidence of pyometra is very low, i.e. <0.01 %. This manuscript describes a case of a 65-year-old postmenopausal patient with pyometra.

## Case presentation

A 65-year-old female patient presented with a history of excessive, foul-smelling white discharge from the vagina, itching over the perineal region, lower abdominal pain, and postmenopausal bleeding for two to three months. Her general and systemic examinations were within normal limits. Her per-vaginal examination revealed an endophytic cervical growth involving the anterior vaginal wall and parametrium bilaterally. Her per-speculum examination revealed fragile necrotic cervical growth, which bled on touch with foul-smelling discharge.

**Figure 1:**
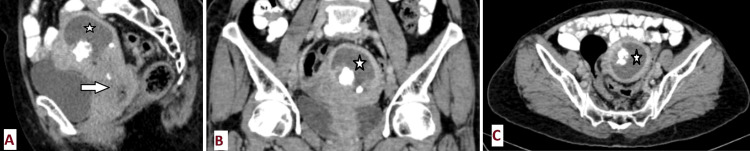
CECT abdomen and pelvis sagittal (A), coronal (B), and axial (C) sections showing heterogeneously enhancing cervical growth (arrow) involving the lower half of the uterus and the upper half of the vagina with resultant endometrial collection (star) and multiple fibroids with calcific degeneration. CECT: Contrast-enhanced CT

**Figure 2:**
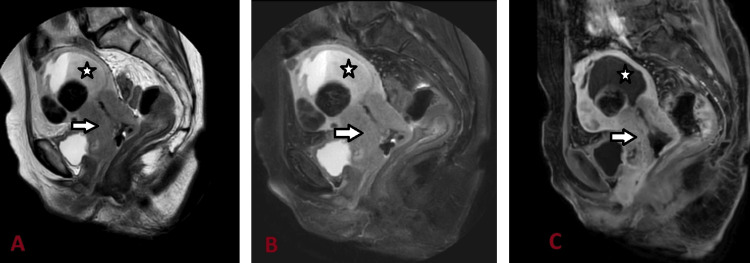
MRI abdomen and pelvis T2 (A), T2-FS (B), and T1-C+ sagittal sections showing heterogeneously hyperintense cervical growth (arrow) involving the lower half of the uterus and the upper half of the vagina with heterogeneous enhancement (C) and resultant hyperintense endometrial collection showing fluid-fluid level (star) and multiple fibroids with calcific degeneration. FS: Fat saturated; C+: Contrast

**Figure 3:**
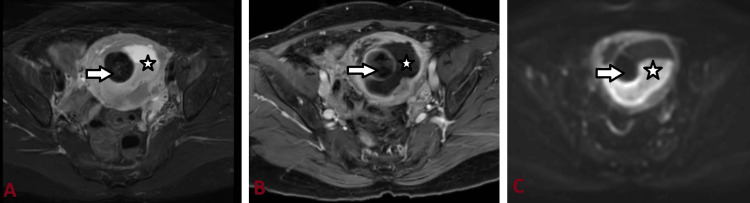
MRI abdomen and pelvis T2-FS (A), T1-C+ (B), and DWI axial sections showing heterogeneously hyperintense endometrial collection (star) and restricted diffusion in DWI (C) suggestive of pyometra. A submucosal fibroid (arrow) can also be seen adjacent to the endometrial collection. FS: Fat saturated; C+: Contrast; DWI: Diffusion-weighted imaging

Her pelvic ultrasound examination was done outside, which revealed heterogeneous ill-defined cervical growth with endometrial fluid collection and multiple uterine fibroids. CT abdomen-pelvis (Figure 1) and MRI pelvis (Figures 2-3) were done in our hospital, which revealed an ill-defined heterogeneously enhancing cervical growth with heterogeneous endometrial collection showing restricted diffusion on MRI (Figure 3) suggestive of pyometra. The growth was extending and involved the lower half of the uterus superiorly, the upper half of the vagina inferiorly, the posterior wall of the urinary bladder anteriorly, and the mesorectal fascia posteriorly with loss of fat planes with the rectum. Bilateral parametrium involvement and loss of fat planes with bilateral lower ureters were noted. Multiple intramural, subserosal, and submucosal fibroids were also noted, with a few of them showing calcific degeneration. Multiple heterogeneously enhancing lymph nodes were noted in the bilateral external iliac region.

**Figure 4:**
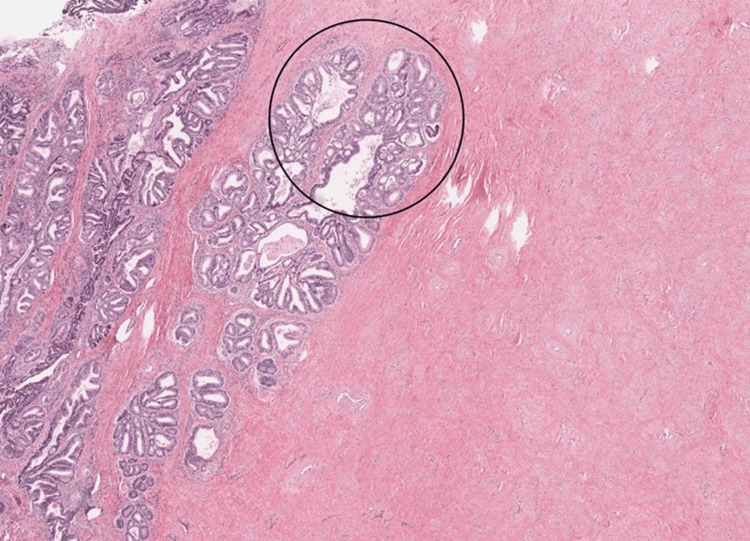
H&E, 20x section showing moderately differentiated cribriform to papillary architecture with haphazard extension of glands (black circle) and stromal reaction in the form of edema and chronic inflammation.

**Figure 5:**
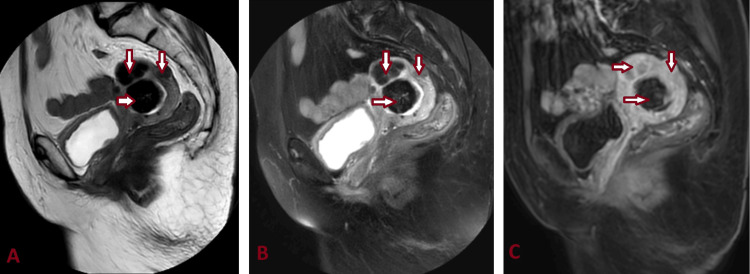
MRI abdomen and pelvis T2 (A), T2-FS (B), and T1-C+ (C) sagittal sections showing no evidence of any focal lesion or significant endometrial collection suggestive of complete resolution. Multiple uterine fibroids (arrows) with calcific degeneration can be seen. FS: Fat saturated; C+: Contrast

She was started on empirical antibiotics, and her cervical biopsy was done that revealed histopathological features (Figure 4) suggestive of moderately differentiated squamous cell carcinoma. She was then referred to our cancer hospital, where she underwent six cycles of chemotherapy. Later, she came for a follow-up MRI scan (Figure 5), which showed no obvious evidence of any focal lesion or significant endometrial collection suggestive of a complete response.

## Discussion

Pyometra is a rare gynecological condition with an incidence of <0.01 % that should be included as a differential diagnosis in postmenopausal women who present with vaginal discharge and abdominal pain. It is most commonly seen in postmenopausal women and postpartum. It can also be seen in younger patients with diabetes, cervical anomalies, cervicitis, large uterine fibroids, endocervical malignancy, recent vaginal surgery, or injection drug use [7,8]. Pyometra develops due to cervical stenosis, which occurs due to thinning and atrophy of the cervix. However, endocervical malignancy should also be kept in mind. Cervical stenosis can also develop in women with cervical malignancy due to the formation of fibrotic scar tissue after certain procedures like brachytherapy, electrosurgical procedures, radiofrequency ablation, or cone biopsy [9].

Pyometra is often diagnosed incidentally [10]. It is caused by different microorganisms, the most common being *Streptococcus, Bacteroides fragilis, and E. coli* [11], leading to the buildup of purulent fluid within the endometrial cavity. Patients present with clinical symptoms like abdominal pain, foul-smelling vaginal discharge, itching in the perineal region, and fever with chills. It is assumed that pyometra is secondary to endocervical malignancy until proven otherwise [12]. The buildup of the purulent material increases the intrauterine pressure, which can lead to uterine rupture if left untreated. Therefore, prompt diagnosis and treatment are required to prevent complications like peritonitis, hemorrhagic shock, fever, and bleeding due to spontaneous uterine rupture.

Pyometra is diagnosed using microbiological or radiological investigations. Transabdominal pelvic ultrasound is the initial radiological investigation. Heterogeneous fluid collection with echogenic debris is seen on ultrasound. Higher imaging modalities like CT and MRI are used to rule out complications. On contrast-enhanced CT (CECT), peripherally enhancing heterogeneous endometrial collection with an air-fluid level can be seen. Intraperitoneal free fluid or free air is seen in case of uterine rupture. On MRI, the purulent endometrial collection appears hypointense on the T1 weighted image (T1WI) and hyperintense on the T2 weighted image (T2WI), showing restricted diffusion on diffusion-weighted imaging (DWI).

Treatment of pyometra includes broad-spectrum antibiotic therapy to prevent peritonitis and sepsis, followed by cervical dilatation and drainage of the purulent collection. Surgical intervention is necessary in post-menopausal women due to high suspicion of endocervical malignancy [13]. In case of spontaneous rupture, the patient has to undergo peritoneal lavage and drainage, emergency laparotomy, or hysterectomy.

## Conclusions

We have discussed a case of pyometra in a 65-year-old postmenopausal woman who presented with excessive, foul-smelling white discharge from the vagina, itching over the perineal region, lower abdominal pain, and postmenopausal bleeding. Ultrasound is the initial imaging modality, which revealed a heterogeneous cervical mass with resultant endometrial fluid collection and multiple uterine fibroids. CT and MRI revealed a heterogeneously enhancing ill-defined cervical mass involving the lower half of the uterus and the upper half of the vagina with resultant endometrial collection showing restricted diffusion on DWI suggestive of pyometra.

Pyometra is a rare cause of abdominal pain in post-menopausal women that is frequently overlooked. Therefore, it should always be included in the differential diagnosis. Malignancy should always be considered as the primary etiology until proven otherwise. Early diagnosis and treatment is necessary to prevent complications like uterine rupture and peritonitis.
